# Dancing Intercorporeality: A Health Humanities Perspective on Dance as a Healing Art

**DOI:** 10.1007/s10912-017-9502-0

**Published:** 2017-12-20

**Authors:** Aimie Purser

**Affiliations:** School of Sociology & Social Policy, University of Nottingham, Room B38, Law & Social Sciences Building, University Park, Nottingham, NG7 2RD UK

**Keywords:** Merleau-Ponty, Phenomenology, Dance

## Abstract

As a contribution to the burgeoning field of health humanities, this paper seeks to explore the power of dance to mitigate human suffering and reacquaint us with what it means to be human through bringing the embodied practice of dance into dialogue with the work of the French philosopher Maurice Merleau-Ponty. Merleau-Ponty’s conceptualisation of subjectivity as embodied and of intersubjectivity as intercorporeality frees us from many of the constraints of Cartesian thinking and opens up a new way of thinking about how dance functions as a healing art through its ability to ground and reconnect us with self, world, and others--with our humanity. It is argued that through a Merleau-Pontian framework, we can come to appreciate the true potential of dance as a positive and deeply humanising experience, demonstrating how expressive arts practice understood through the lens of philosophical theory can open up new dimensions of understanding and experience in relation to well-being and self- (and other-) care.

## Introduction

Dance plays a role in healing rituals across a number of cultures and is also recognised to promote social bonding (Chaiklin 2009). Indeed even in contemporary Western medicine, dance is used in psychotherapeutic contexts in the form of dance/movement therapy (DMT). As a contribution to the burgeoning field of health humanities, this paper seeks to explore the power of dance to mitigate human suffering and reacquaint us with what it means to be human through bringing the embodied practice of dance into dialogue with the work of the French philosopher Maurice Merleau-Ponty.

The promise of the health humanities – and we should note here that the medical/health humanities should not be thought of as a discipline but rather as a multidisciplinary, interdisciplinary or perhaps even post-disciplinary field of study (Atkinson et al. 2015) – is of a broader and richer understanding of what is healthful and therapeutic through exploration of and insight into the human condition. As such, it celebrates the uses of arts and humanities within traditional healthcare settings, practices and training and calls for a reimaging of the boundaries of health and healing, so that our intellectual and therapeutic focus might escape the physical and, perhaps more importantly, the epistemological constraints of the clinical (Crawford et al. 2010; Crawford et al. 2015; Jones et al. 2014). In this spirit, this article presents an alternative understanding of dance as therapeutic, which is based in philosophy rather than in the psy-disciplines or the neuroscientific insights that currently dominate the literature of DMT as a clinical practice.

Of particular significance for an alternative framing of dance is Merleau-Ponty’s conceptualisation of human being – as embodied subjectivity or the ‘body-subject’ - in a way that transcends the traditional Cartesian dualist distinctions between mind and body and between subject and object (see Purser 2011). While DMT is clearly a bodily/physical practice used in a mental health or psychotherapeutic intervention, the psy- (and sci-) disciplines which currently underpin expressive arts therapy tend to remain locked in a Cartesian framework which struggles to fully integrate our bodily exterior with our psychic interior. The focus on the content of artistic expression as a route to the unconscious and on the co-production of art or movement as a route to a therapeutic relationship means that creative practice is conceptualised as a way to facilitate verbal dialogue, as the true medium of therapy (Skaife 2001).

In contrast to this, Merleau-Ponty’s non-dualist philosophy, in its illumination of the bodily basis of being, allows us to make sense of the embodied creative practice of dance as therapeutic in its own right. Thus, in line with David Pilgrim’s recent reflections on the philosophical aspirations of the medical humanities, not only does this approach ‘create an opportunity to provide a richer epistemology,’ but it also allows for a ‘more sensitive approach to ontology than medical positivism’ (2016, 442). In locating subjectivity in the lived body, Merleau-Ponty locates our capacities for thinking and feeling in a sensual and sensuous body which is embedded in and oriented towards the world around us and the others in our environment. Contra Descartes, there is no separate realm of the mental in which thinking occurs; only embodied beings making sense of the world they inhabit. Our thoughts are manifest through the body, be it in language or gesture, and in the world, be they silent, vocalised or expressed through the creative arts (Merleau-Ponty 1964a, 1964b).

Importantly for Merleau-Ponty, our thoughts come into being in a world which, as embodied beings, we are directly part of and also share with others. We are thus presented with an understanding of human being which orients us towards thinking in terms of a shared world where thoughts come into being through bodily expression and where intersubjective relations have to be understood as taking place between situated embodied beings. In focussing on this shared world and on the corporeal dimensions of intersubjectivity – intersubjectivity as *intercorporeality* – Merleau-Ponty frees us from many of the constraints of Cartesian thinking and opens up a new way of thinking about how dance functions as a healing art through its ability to ground and reconnect us with self, world, and others—with our humanity.

This paper will proceed by elaborating the concept of intercorporeality through a detailed explication of Merleau-Ponty’s thought before exploring how this plays out in the embodied practice of dance. In order to attend to the experience and the potential of dance, this study draws on in-depth qualitative interview accounts of sixteen professional contemporary dancers from United Kingdom (UK) repertory companies. In keeping with the aims of health humanities to broaden our understanding of the healthful dimensions of the arts and to democratise our approach to the arts in health- (and self-) care beyond the clinical setting, this is not, therefore a study of DMT therapists and patients; rather it is a study of individuals for whom dance forms a central part of their lives. Professional dancers, in particular, were chosen for this study because their daily engagement with dance as reflexive practitioners gives them a heightened awareness of their own embodiment and that of others.

## Theoretical underpinnings

For Merleau-Ponty the primary sense of self is not understood in the Cartesian dualist sense which conceptualises human subjectivity in terms of *res cogitans* (thinking substance) while rendering the body mere *res extensia* (extended or physical substance). Rather, prior to Descartes’ *cogito* – ‘I think therefore I am’ – there is the ‘tacit *cogito*’ – ‘I can’ – the feel we have of our body and how it connects us to the world. Merleau-Ponty (2002) also denotes this pre-reflective feel that we have for our body’s positioning and possibilities for action with the term, corporeal schema. My corporeal schema is thus my primary sense of self or ‘I’ in the sense of the ‘I can.’

While the Cartesian tradition has struggled with the problem of intersubjectivity, also known as the problem of ‘other minds,’ Merleau-Ponty’s rethinking of self as situated and embodied opens up the possibility for rethinking self-other relations. Descartes’ *cogito* sets up both what it is to be a conscious subject and what it is to know a conscious subject so that the only way to know a conscious subject is to be that conscious subject reflecting on itself: ‘I think therefore I am.’ Thus where human being is defined, as per Descartes, as *res cogitans*, I can only have access to myself and can never be sure of the existence of other human beings. This situation of solipsism – the isolation of the subject from others – is arguably intrinsic to and insoluble within Cartesian thought.

In contrast to this, Merleau-Ponty shifts the focus from the private, invisible experience of thought to the lived body through his redefinition of human being in terms of embodiment and behaviour, these being visible and publicly available. If self or subjectivity is not non-material consciousness but is rather manifest in my animate embodiment, then we do not have transparent access to self through introspection as Descartes suggests but rather achieve self-knowledge through action. The Cartesian problem of solipsism is thus dissolved in Merleau-Ponty’s framework as my existence as a self comes into being and comes to my awareness in the same shared world where other selves are coming into being and to my awareness. I do not have complete, transparent access to myself, but neither is my knowledge and surety confined to an inner realm of consciousness: if to be human is to be a human body and exhibit human behaviour then, as Dillon suggests in his book *Merleau-Ponty’s Ontology*, ‘I can see your humanity and you can see mine’ (1997, 113).

Intersubjectivity, for Merleau-Ponty, is therefore based in a mutual awareness, which is understood as a reciprocity of perception. This reciprocity is encapsulated in the concept of reversibility which becomes a motif throughout Merleau-Ponty’s writings being most fully explored in his later work including his last published essay *Eye and Mind* (1964a), which focuses on visual art and the embodiment of the painter in the world and his incomplete final work *The Visible and the Invisible* (1969). Reversibility is primarily conceptualised through the basic model of one of my hands touching the other where the hand that touches can also be touched by the hand that was originally touched but is now touching. Indeed being able to touch anything requires that the toucher is also touchable, as to touch a thing is to feel the thing touching me.

This perspective dissolves any clear distinction between touching and touched, sentient and sensible, and thus between the body as subject and the body as object. I experience my body as neither wholly Cartesian subject – in which case it would be invisible – nor as wholly object – in which case it would not be able to serve my intentions. Indeed object manipulation requires that I both see my hand in relation to the object I wish to grasp and that this wish or intention to grasp is lived through my hand (Merleau-Ponty 1964c). Thus I understand and inhabit my body as simultaneously part of my intentional subjectivity and as an object in the world – an awareness that Merleau-Ponty calls corporeal reflexivity. This corporeal reflexivity allows that there is overlap, not only between my body as subject and my body as object but also between my experience of my (visible and touchable) body and my experience of other bodies, explaining the possibility of imitating the behaviour of the Other despite the fact that the outer look of the behaviour is not the same as the inner feel of the behaviour. As Dillon explains:

My body is the ground of my identity for myself, hence it can function as the ground of my identity for others, and your body plays the same role for you, me, and the others who dwell in our world. Furthermore, the isomorphism of our bodies provides a basis for mutual understanding: I understand the behaviour of your hands as I see them from the outside because my hands are similar to yours and I know them from the inside. (1997, 113-114)

Thus there is a reversibility to intersubjective relations which relies on my own sensibility as well as my sentience: the Other and I are mutually available to each other through our perception of each other. The corporeal schema—my primary, embodied sense of self— is not a private, inner realm but rather is visibly and tangibly manifest in my embodied behaviour, and as embodied, sentient and sensible beings open to a shared world, we experience each other in what Merleau-Ponty terms carnal intersubjectivity or *intercorporeality*. This intercorporeal connection with the Other is referred to as transfer of corporeal schema, and it is through this process that we recognise other human beings as like ourselves, making it the grounds of intersubjectivity.

## Approach/methods

Merleau-Ponty’s philosophy emphasises the importance of a tacit or pre-reflective sense of self which precedes or underlies the reflective thought involved in the Cartesian *cogito*. Understanding our subjectivity and what it is to be human, thus requires attending to our pre-reflective sense of ourselves as embodied beings in the world. Direct experiences of dance are particularly helpful for this because as an embodied practice and an expressive art, engaging in dance focuses us on our bodily presence in the world and on the here-and-now rather than encouraging detached reflection. As Maxine Sheets-Johnstone (1981) describes, dancing involves a thinking *in movement* rather than a thinking about movement; body and intentional being are one. Thus in dance we develop our awareness of what Merleau-Ponty refers to as the corporeal schema, our immediate tacit sense of embodied self, which dancers in this study described with the terms ‘being-in-your-body’ and ‘being-in-the-moment.’

The importance of focusing on the present moment or the here-and-now has already been identified as significant for psychotherapeutic progress because of its ability to facilitate the therapeutic relationship both in individual psychotherapy and in group therapy (Yalom 1985), and expressive arts therapies including DMT have been recognised as useful because of their ability to produce this focus (Erfer and Ziv 2006). The following discussion of dance, however, departs from the understanding of art practice as a tool for producing material for a psychotherapy session or for facilitating the building of the therapeutic relationship. Rather, in seeking to understand through Merleau-Ponty what it is that occurs when we dance together, this paper makes a case for a philosophically informed position which sees dance not simply as a means to a therapeutic end but as an end in itself in terms of its ability to meaningfully and directly (re-)engage us with self, others and our environment—to bring us back to our humanity. As such it is in tune with the aim of the medical health humanities not to make sense of the arts from within the framework of the biomedical world-view but to ‘refocus the whole of medicine in relation to an understanding of what it is to be fully human’ (Greaves and Evans 2000, 1).

The data drawn on in this section comes from a qualitative interview study which explored the experience of dance and being a dancer. Dancers were recruited to the study through their places of work, and the data presented in this paper is from a convenience sample of sixteen professional dancers at two separate repertory companies in the United Kingdom. Ethical clearance was sought from the University of Nottingham prior to any contact with participants, and full informed consent procedures were implemented with each participant before interviews began.

During the interviews I asked each interviewee the broad question of what it was like to dance with someone in the context of conversation about fairly mundane aspects of dance, revolving around issues like learning new material and questions about the practice and process of dance such as how they knew what to do next and how they knew when they had learnt a choreography. Such questions were intended to tap into dancers’ reflections on the pre-reflective or taken-for-granted aspects of their embodied practice and to encourage further reflection on any areas that their previous training and practice had not already led them to reflect on. This mundane focus on practice was also a key and productive part of the conversations about dancing with others where dancers were able to talk about tacit and pre-reflective aspects of intersubjective negotiation and understanding. This topic was, however, also the one which produced the most lengthy and poetic responses from my interviewees, as can be seen in some of the quotations below, where dancers brought in new dimensions such as talk of the soul and intimacy that did not arise in answers to my other questions.

The following discussion uses a Merleau-Pontian framework to make sense of the mundane and physical aspects of dancing together and also the spiritual or transcendent aspects of the experience, not as separate dimensions but as inherently linked. It is only through an understanding of our fundamental groundedness in our bodies that we can fully understand the moments of interpersonal synchrony, connection, intimacy and understanding that are central to our experience of intersubjectivity and are so beautifully illustrated in the practice of dance.

## The experience of dancing with others

The process of joint movement, of dancing with someone else, is particularly interesting for rethinking intersubjectivity in Merleau-Pontian terms as it involves a form of connection or communication which is achieved without words and through the medium of bodily contact. As Louisa suggests, openness to such bodily communication is part of an overall tacit or pre-reflective awareness that the dancer has of her embodiment and situation within the immediate context of the dance:

*If you’re in the moment and you’re on stage and you’re aware – you’re in the moment and you’re in your body, you’re in that part of the piece, but you also have to be super-aware in the way that you’re ready to accept anything, and that’s like that communication that happens which is not, you don’t talk you just know, you, you even feel it in, you feel inside and you just react – that’s the strange thing and that’s really exciting when you just have that, when it’s in sync like that. [Louisa]*

Here the dancers are not consciously formulating thoughts or reflecting on the situation but are reacting to each other – to each other’s bodies – at a pre-reflective level. Louisa describes the direct response she experiences to the Other’s movement as the two dancers being ‘in sync’ and emphasises that she knows or feels how to move with or respond to the Other with an immediacy which does not involve discussing or reflecting on the process.

Thus dance grounds us in our own body-subjectivity or bodily intentionality and also orients us to or opens us towards the body-subjectivity or bodily intentionality of the other dancer. The analogy with conversation used by many of the dancers is significant because this notion of a (tacit or unspoken) dialogical interaction emphasises a two-way process between two mutually engaged beings. Dancing together thus involves a reciprocal openness or awareness allowing this type of tacit bodily communication to occur:

*There’s this like different kind of awareness that you have to have, just because you have to be able to move together, in a small space, and big space, so em, you definitely have to have that self-awareness and knowing kind of, kind of not just being taking care of yourself but I think what is the nicest part as well when you do get to dance actually with someone, … you have to talk with your bodies so you have to kind of listen to each other – you can’t always do it your way, you have to find the way. [Anna]*

The awareness of and connection with other dancers achieved in this way is not just therefore, limited to understanding the materiality of their bodies in terms of weight and position in space but also includes an understanding of them as intentional beings who want to do things in certain ways that may be different to what you want. The dancers are able to recognise each other through their bodily interaction as other physical objects in the world and as other body-subjects, and it is this capacity which allows for the claim that dance enhances our connection with our embodied selves but also with the Other as Other. The dancer does not simply subsume the presence of the Other into her own perspective on the world but is drawn out of herself to recognise the Other’s situation and intentions and experience genuine communication and expressive collaboration.

Tara again here makes reference to the notion of talking and listening through the body when working with a new partner:

*You can tell a lot without, even just working closely with them, just from the look or the way they, their body works with yours, and how they, you can kind of listen to each other through your bodies. You can become quite close to people – you have to be prepared to work very closely with people physically, but because you’re so close physically you, it opens up something mentally as well, there’s some connection there. [Tara]*

Tara’s comments about the link between physical closeness and mental closeness can be understood in terms of the Merleau-Pontian notion of transfer of corporeal schema where we can come to know people’s thoughts, feelings and intentions through identifying with them at the level of the corporeal schema. It is through this pre-reflective intercorporeal identification that I can immediately sense, for example, sadness or anger in the Other, but this facility is heightened and extended in the case of dancers who work closely with each other over time and whose interpersonal connections are formed in the context of joint artistic expression.

Dancers primarily learn dance through processes of imitation, mirroring or copying others’ movements, which can be understood as involving intercorporeal overlap between how the dancer experiences his or her own body and how the dancer experiences the body of the person demonstrating the movement. In addition to this, much new choreography is developed through processes of contact improvisation where the dancers spontaneously move together allowing patterns of movement to emerge from their mutual contact rather than being conceptualised beforehand. Dance training and its creative practice therefore open us up to the reversibility inherent in intercorporeal relations, and Tara’s comments suggest that the intercorporeal identification involved in learning dance also makes the dancer more open to those dimensions of the Other’s existence that she describes as mental.

Intercorporeality and transfer of corporeal schema is thus enhanced in dance, allowing dancers to come to understand other dancers physically in terms of how they move and to develop a sense of closeness, connection or communion at a human, mental or emotional level when dancing with someone. Through moving with each other and attending to the corporeal schema of the other dancer, dancers can come to understand and experience a kind of physical and emotional or mental synchrony, a kinaesthetic empathy, with the dancer with whom they are moving. Dancing with another person thus returns us to a recognition of our shared humanity and our capacity for mutual openness and connection.

Indeed my interviewees emphasised that dance is characterised by mutual openness in the sense of both awareness and honesty:

*You do get to know somebody then because you get to see em, – it’s really difficult to explain, but you get to see them for who they are, you know because people have a lot of barriers and a lot of masks upon themselves a lot of the time and if you’re really invested into the moment and invested in this connection then you have to let those masks and those barriers fall down so that you can feel one another, be with one another and experience this thing with one another and I mean, when it gets to that point you know that person whether they’re feeling sad or whether they’re feeling happy and they don’t even have to even say anything so you know, you have a sense of how they are that day and you take that into account - there’s not a judgement on that it’s just this is how the person is today, this is how I am today and this what it is today and that’s why it’s beautiful. [Steven]*

This is not to say that dancers do not communicate with each other verbally. There was, however, a clear suggestion from all my interviewees that being physically close, ‘in tune,’ or synchronised with another dancer allowed some access to the thoughts and feelings of the Other without anything being reflected on or said. For Merleau-Ponty it is this direct openness to others and to a shared world which characterises intercorporeality, and in the above quote Steven emphasises that the expressive qualities of the two dancers are absorbed into the dance on a particular day without anyone reflecting on or judging the quality of the Other’s movement. The process of creative collaboration thus allows the dancers to experience their own and their partner’s mood as jointly expressed in the co-created movement of dancing together.

Steven describes this, somewhat hesitantly, then, as an interconnection or communion of the two dancers’ souls as well as their bodies which he likens to intimacy of the experience of making love:

*I think that you get to know people incredibly well through dancing – incredibly, incredibly well in a way which is really quite beautiful actually, really quite beautiful because it, because, because it, because of the context of it, it allows space for you to – I don’t want to sound really cheesy here – but almost for like your, when it, for your souls to interconnect in many senses because there isn’t the em, sexuality or ego or all these other kind of things placed on top of it, it’s just simply about being with someone in the space and connecting with someone and that is such a beautiful sensation. I mean, I’ve chatted to my friends who are not dancers about this and I think the only way that I often explain to them about how wonderful it is to dance and so on is like imagine, making love to someone but you’re not – d’you know what I mean? – that totally doesn’t make no sense – You’re as intimate with, you’re as connected with that person, you know, and obviously it doesn’t always get to that level but when it does that’s when not only do you feel it but the audience feels it as well – it gets to a place where you’re communicating, you’re operating on a level of sensation and connection and it’s almost like you’re, you’re having a conversation of sensation but there’s no attachments or connotations of anything else really – it’s really quite beautiful, really something quite special.[Steven]*

The comparison he makes with sexual intimacy is interesting, not only because shared sexual or orgasmic experience has been suggested above as one of the most recognisable examples in adult life of a sense of communion or synchrony through transfer of corporeal schema but also because Steven further qualifies his use of this example. Steven in fact suggests that dance is an even better example of this kind of interconnection than sex because he associates sex with notions of egoism and sexuality, which I take to mean that there are more issues around one person having and showing off his power over the Other in sex. In contrast, dance, for Steven, ideally seems to allow for intimacy without these power relations and thus something more akin to communion of the souls. This is unlikely to be true of all sexual encounters and all dancing encounters, but the context of dance as creative collaboration opens up the possibility for us to transcend our individual ego-centric concerns and feel that we are genuinely in touch with the Other in a direct and open process of co-creation and co-expression. It was this ability to come out of oneself and experience mutual connection with the Other which my interviewees talked about as making the experience of dancing with someone else particularly ‘special’ or ‘beautiful.’

In Steven’s words, dance as an expressive art form ‘allows space’ for this type of experience in a way that we do not find in other facets of life. It is not the case, however, that a connection or intimacy of this kind is always established between dancers working together. Anna, for example, mentioned that there were certain dancers in companies with whom she had felt uncomfortable while Carrie, again using conversation as an analogy for dancing with someone, commented of forming a connection with another dancer that:

*You just naturally gel with another person like you would, you know, having coffee, sometimes you do, sometimes you don’t. [Carrie]*

It also appeared that this type of intimacy or syncretism between dancers was more usually established in a company where the dancers worked together closely over long periods of time. Rhianna describes this process as the ongoing negotiation of an unspoken relationship between dancers where:

A*fter you work with someone for a while you get to like, you feel their body, you feel like where they’re going to take your, you just, you know if you’re working on a duet together you just develop that understanding of how much a risk you can take within that and each other. [Rhianna]*

She further describes this relationship as something that is developed through working physically closely together:

*As you start to like get comfortable with it and start to explore the connection, you develop your own story I think without speaking to each other – you know, you don’t say “oh when I’m dancing with you I feel like you’re this and this that and the other” – you just sort of, you don’t even know yourself exactly what, necessarily what the relationship is between you but you do definitely develop something that’s like both of you understand physically but don’t necessarily put into words – I think it’s quite special. [Rhianna]*

In a continuation of the passage quoted above, Steven further explains:

*You do get to know a lot about people when you dance with them because you’re working with them all the time and you sweat – you sweat with one another for goodness sake – you know when you sweat with someone you get to know everything about them … it kind of is so, such a close-knit thing and you have to be so co-dependent, you know, it’s so, you know, it’s impossible for you not to get to know someone really well. [Steven]*

Here, again, Steven’s account of dancing with someone emphasises notions of mutual openness, and both Rhianna and Steven evoke a sense of vulnerability in their use of terms such as risk and dependency. What is special about the relationship formed when we dance with another person is therefore that it develops in us a capacity for openness towards the Other which may feel too dangerous in alternative situations where it doesn’t arise pre-reflectively from mutual trust being slowly built up in the process of joint movement. It provides a context in which mutual openness (and its attendant vulnerability) develops between embodied beings, returning us to an understanding of our basic potential to connect with the Other and the world and thus with our own humanity.

## Conclusion: dance, health and humanity

In a world where negative feelings of detachment, fracture and alienation have consistently been identified by psycho-social theorists as ‘symptoms’ of modern living, it becomes increasingly important that any understanding of health engages with issues of groundedness and connectedness. In the spirit of the health humanities, this paper has offered the philosophy of Merleau-Ponty as a basis from which we might begin to make sense of our being-in-the-world as body-subjectivity and also of our connection to others as embodied and as borne of our mutual situatedness in a shared world, thus opening up a different kind of conversation about the therapeutic value of dance from those generally found in the psy- and sci-informed disciplines with their Cartesian underpinnings.

Importantly, it is dance as an end in itself that is brought centre-stage in this discussion, and the focus on the experiences of those who engage in dance as creative practice rather than those who subsume dance into their broader (psycho-) therapeutic practice is significant for re-adjusting the way we think about dance (and all creative arts) as healing and life-enhancing. This move out of the clinical setting is also significant in the context of the aims of the health humanities to democratise the practice of healing arts beyond professions such as DMT and to extend their reach beyond patient populations.

As has been shown in the discussion above, dance stimulates a particular kind of awareness that not only helps us to experience ourselves in a more holistic way as embodied beings by grounding us ‘in the moment’ and ‘in your body’ but also opens us to a direct connection with the embodied Other. Here, dance allows us to experience a form of communication or dialogue with the Other characterised by a mutual openness and a transcendent state where self and other are both drawn out of themselves into the ongoing communicative and creative experience of co-expression. Through this Merleau-Pontian framework we can come to appreciate the true potential of dance as a positive and deeply humanising experience, thus demonstrating how expressive arts practice understood through the lens of philosophical theory can open up new dimensions of understanding and experience in relation to well-being and self- (and other-) care.
